# Nondestructive measurement of kiwifruit firmness, soluble solid content (SSC), titratable acidity (TA), and sensory quality by vibration spectrum

**DOI:** 10.1002/fsn3.1390

**Published:** 2020-01-20

**Authors:** Wen Zhang, Aichen Wang, Zhenzhen Lv, Zongmei Gao

**Affiliations:** ^1^ School of Life Science and Engineering Southwest University of Science and Technology Mianyang Sichuan PR China; ^2^ School of Agricultural Equipment Engineering Jiangsu University Zhenjiang Jiangsu PR China; ^3^ Department of Biological Systems Engineering Washington State University Prosser WA USA

**Keywords:** firmness, impact, kiwifruit, maturity, nondestructive measurement

## Abstract

Maturity is a key attribute to evaluate the quality and acceptability of fruit products. In this study, the impact method was used for nondestructive measurement of kiwifruit maturity. The fruit was vertically dropped onto an impact plate, and an accelerometer was used to measure the response signal. Then, fruit firmness, soluble solid content (SSC), titratable acidity (TA), and sensory scores were measured to determine the kiwifruit maturity. In addition, different modeling methods were proposed for data analysis. The results showed that the optimized prediction results were obtained by the principal component analysis–back‐propagation neural network (PCA‐BPNN) method for both quantitative and qualitative analysis. The optimized correlation coefficient between prediction and actual values (*r*_p_) and root mean square error of prediction (RESEP) for firmness, SSC, TA, and sensory score were 0.881 (2.359N), 0.641 (1.511 Brix), 0.568 (0.023%), and 0.935 (0.693), respectively. The optimized discriminant accuracy for immature, mature, and overmature kiwifruits was 94.2% and 92.1% for calibration and validation, respectively. Such results indicated the feasibility of the proposed impact method for kiwifruit maturity evaluation.

## Graphical abstract

The fruit was vertically dropped onto an impact plate, and an accelerometer was used to measure the response signal. The results showed that best prediction results were obtained by the principal component analysis–back‐propagation neural network (PCA‐BPNN) method for both quantitative and qualitative analysis. Good performance of the prediction models proved that the proposed impact method for kiwifruit maturity evaluation was feasible.

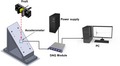

## 1 INTRODUCTION

Kiwifruit is a typical kind of climacteric fruit (Antunes & Sfakiotakis, 2000). Kiwifruit keep softening during its ripening process and storage. The maturity is quite important for the evaluation of quality and acceptability of kiwifruit product. Accurate assessment of kiwifruit maturity can help both consumers and distributers to determine its harvest time, quality, and storage potential (Mayorga‐Martínez, Olvera‐Trejo, Elías‐Zú Iga, Parra‐Saldívar, & Chuck‐Hernández, 2016; Taniwaki, Hanada, & Sakurai, 2009).

However, the traditional methods are usually time‐consuming and costly. Moreover, these methods are destructive and only can be used for sampling inspection. Therefore, various nondestructive technologies were proposed for fruit maturity evaluation, including the near‐infrared (NIR) spectroscopy (Alhamdan & Atia, 2017), ultrasonic method (Mizrach, 2004), magnetic resonance imaging (Zhang & McCarthy, 2012), machine vision (Payne, Walsh, Subedi, & Jarvis, 2013), electronic nose technique (Hernández Gómez, Wang, Hu, & García Pereira, 2007), and acoustic vibration method (Mayorga‐Martínez, Olvera‐Trejo, Elías‐Zúñiga, Parra‐Saldívar & Chuck‐Hernández, 2016).

Among these nondestructive methods, the acoustic vibration method has been proved to be an effective way for fruit maturity evaluation, especially for the climacteric fruit. The contact and noncontact measurements were two main technologies in the acoustic vibration method (Taniwaki & Sakurai, 2010; Zhang, Lv, & Xiong, 2018). The acceleration pickup (De Belie, Schotte, Coucke, & De Baerdemaeker, 2000) and piezoelectric sensor (Macrelli, Romani, Paganelli, Sangiorgi, & Tartagni, 2013) were usually used in the contact measurement. However, the attachment of contact sensor to the fruit would affect the free vibration of tested sample, and even damage the surface of fruit (Zhang et al., 2018). Therefore, contact sensors were seldom used in the online detection. The noncontact measurement was getting more and more attention. The microphone was one of the most commonly used noncontact sensors (Valente, Leardi, Self, Luciano, & Pain, 2009). However, the microphone was easily affected by the ambient noise. The laser Doppler vibrometer, as an optical detector, was another commonly used noncontact sensor (Zhang, Cui, & Ying, 2014). A problem of laser Doppler vibrometer was its high price.

In some existing studies, the contact sensor was attached to an impact plate for the indirect measurement of fruit, which did not affect the tested sample and was low cost (Hosainpour, Komarizade, Mahmoudi, & Shayesteh, 2011; Ragni, Berardinelli, & Guarnieri, 2010). Therefore, the impact method by dropping the fruit onto an impact plate was used in this study. An accelerometer was attached to a specially made impact plate rather than the tested sample. The vibration response was different obtained from the samples with different maturities. Moreover, the noncontact merit can meet the requirement of online detection. Similar devices were used for the detection of potato and fruits (Hosainpour et al., 2011; Ragni et al., 2010). However, a problem of such design was that the impact between the sample and plate may damage the tested sample. Therefore, different drop heights were analyzed for seeking the optimized value in this study. In addition, different modeling methods were proposed for data analysis. The study aimed to investigate the feasibility of the proposed impact method for the quantitative and qualitative analysis of kiwifruit maturity.

## 2 MATERIALS AND METHODS

### 2.1 Kiwifruit samples

Kiwifruit samples (*Actinidia deliciosa*. cv. “Hayward”) were harvested about 160 days after flowering from a local orchard and immediately transported to the laboratory at Southwest University of Science and Technology in Mianyang, China. The fruits were stored in the laboratory at a temperature of approximately 20°C and a relative humidity of approximately 60% for 20 days. Fruits that spoiled during storage were removed, and a total of 217 fruit samples were finally used for the experiment. Table 1 shows the basic morphological properties of the tested kiwifruit samples.

**Table 1:** Morphological properties of the tested kiwifruit samples

	Average	Maximum	Minimum	Standard deviation
Mass (*m*, g)	124.47	171.17	88.53	26.34
Height^a^ (*h*, mm)	58.07	67.10	49.78	4.63
Diameter^a^ (*d*, mm)	52.25	58.78	46.56	3.78

a Average value of three measurements taken at evenly spaced interval of 120°.

### 2.2 Measurement of impact response of kiwifruits

**Figure 1:**
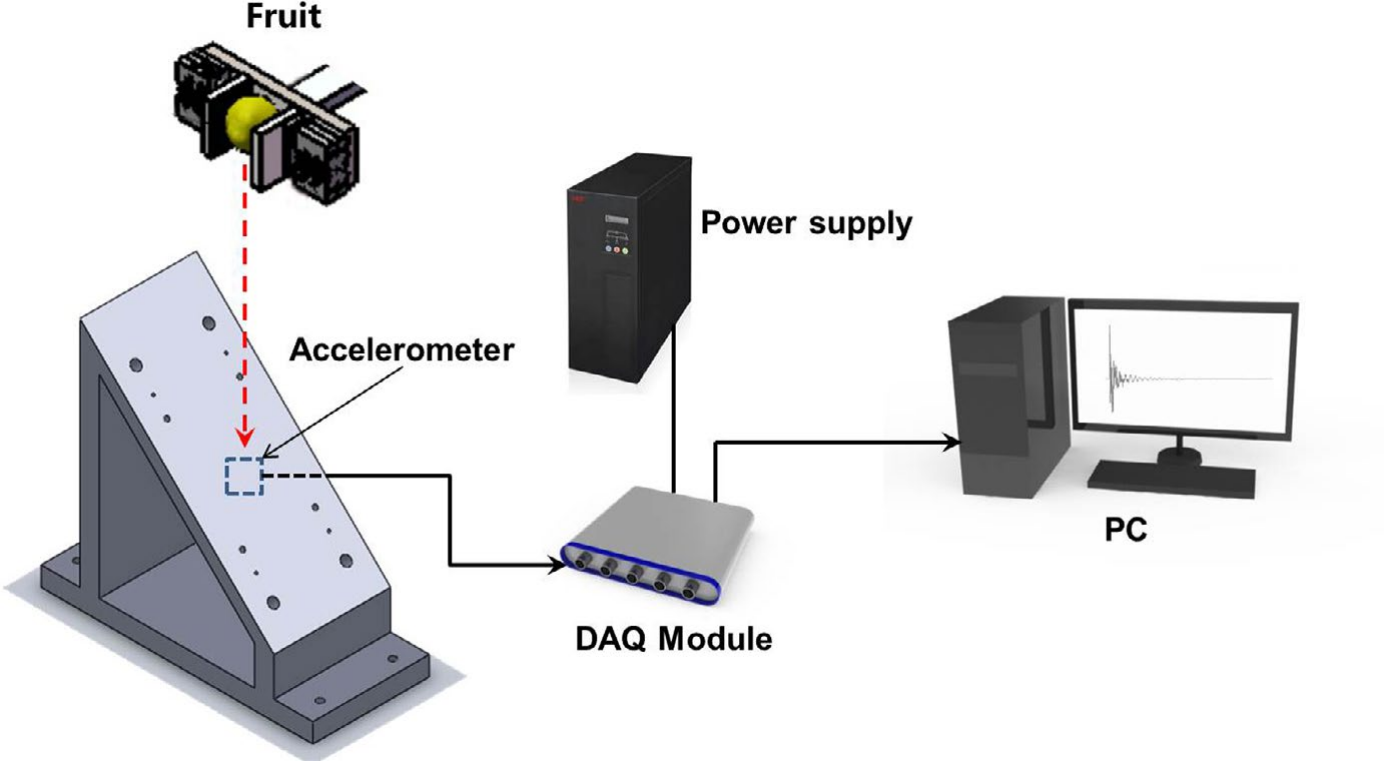
Schematic diagram of experimental setup for measuring the impact response of kiwifruits

A schematic diagram of the experimental setup used to measure the impact response of kiwifruits is shown in Figure 1. The system consisted of an aluminum impact plate (22 × 12 × 2 cm), a pneumatic manipulator (HZ5014; Lixian Instrument Co., Ltd.), an accelerometer (KT1010; Baofei Vibration Instrument Co., Ltd.), a data acquisition (DAQ) module (DFT; Baofei Vibration Instrument Co., Ltd.), and a PC. The fruit with stem–calyx horizontal was vertically dropped onto the impact plate fixed on an optical table by a pneumatic manipulator from the height of 2, 4, and 6 cm. The impact plate has a 45‐degree inclination to avoid a second impact between the fruit and impact plate. The falling distance and angular were determined by a preliminary experiment. The accelerometer was attached on the middle of the back of the impact plate for signal acquisition. Then, the response signal was acquired by the DAQ module and delivered to the PC. The data acquisition software was DFT600 (Baofei Vibration Instrument Co., Ltd.).

**Figure 2:**
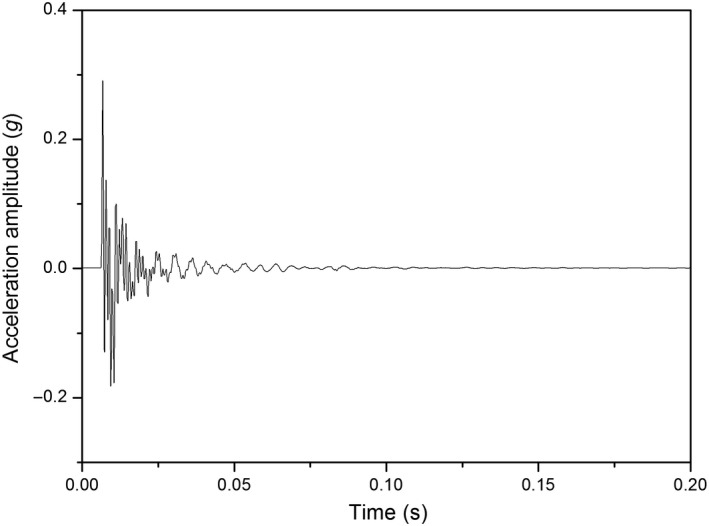
A typical impact response signal of kiwifruit

Figure 2 shows a typical impact response signal of kiwifruit. The trigger value of accelerometer for data collection was 0.01 *g* (*g* = 9.8 m/s^2^). The data sampling frequency was 5 kHz, and 1,024 data were collected for each sample. The signal started from the impact between the sample and the impact plate, and gradually decayed to zero caused by oscillation of the impact plate. The extraction of response signal started when the signal value was greater than 0.01 *g*, and ended when the signal value was smaller than 0.01 *g* by an MATLAB procedure.

For each impact response signal, a total of 15 vibration parameters were extracted from the time domain signal, including the mean value, variance, maximum value, minimum value, signal duration, average rectified value, waveform area, root mean square, skewness, kurtosis, peak‐to‐peak value, crest factor, impulse factor, waveform factor, and margin factor (Zhang, Cui, & Ying, 2015). The formulas of some vibration parameters are listed in Table 2.

**Table 2:** Vibration parameters and their formulas^a^

Feature description	Formula	Feature description	Formula
Mean value	$X_{\text{mean}}=\frac{1}{n}\sum_{i=1}^{n}x_{i}$	Variance	$s^{2}=\frac{\sum_{i=1}^{n}{\left(x_{i}-\bar{x}\right)}^{2}}{n-1}$
Average rectified value	$X_{\text{arv}}=\frac{1}{n}\sum_{i=1}^{n}\left|x_{i}\right|$	Waveform area	$A_{w}=\sum_{i=1}^{n}x_{i}\frac{1}{F_{s}}$
Root mean square	$X_{\text{rms}}=\sqrt{\frac{1}{n}\sum_{i=1}^{n}x_{i}^{2}}$	Skewness	$S_{k}=\frac{\sum_{i=1}^{n}{\left(x_{i}-\overset{\_}{x}\right)}^{3}/n}{s^{3}}$
Kurtosis	$K_{u}=\frac{\sum_{i=1}^{n}{\left(x_{i}-\bar{x}\right)}^{4}/n}{s^{4}}-3$	Peak‐to‐peak value	$X_{\text{peak}}=\max\left(x_{i}\right)-\min\left(x_{i}\right)$
Crest factor	*C* = *X*_peak_/*X*_rms_	Impulse factor	*I* = *X*_peak_/*X*_arv_
Waveform factor	*W* = *X*_rms_/*X*_arv_	Margin factor	$M=X_{\text{peak}}/{\left(\frac{1}{n}\sum_{i=1}^{n}\sqrt{\left|x_{i}\right|}\right)}^{2}$

a *x_i_* are the values of the response signal, *n* is the number of data points, and *F_s_* is the sampling frequency.

### 2.3 Maturity evaluation

#### 2.3.1 Firmness

Fruit firmness was measured by the puncture test using a texture analyzer (TA‐XT2i, Stable Micro System, Inc.). A 5‐mm‐diameter cylindrical probe was used to perforate kiwifruits with peel at two opposite sites along the equatorial plane. The penetration speed was 1 mm/s, and the penetration depth was 8 mm. The maximum force during penetration was recorded as fruit firmness.

#### 2.3.2 Soluble solid content

Half of each sample was used to make juice, and 1 ml juice was used to measure SSC (°Brix) with a digital refractometer (PR‐101a, Atago, Co.).

#### 2.3.3 Titratable acidity

Measurement of TA, expressed in percentage of citric acid, was carried out with an automatic titrator (G20, Mettler Toledo).

#### 2.3.4 Sensory evaluation

Half of each kiwifruit was equably cut into 10 parts for 10 trained panelists (Chen & Opara, 2013). Every panelist graded the overall maturity of kiwifruit based on appearance, firmness, sweetness, and juiciness. The maturity of kiwifruits was rated on a scale of 1–10. For qualitative analysis, the kiwifruits with sensory score of 8–10, 5–7, and 1–4 were categorized into immature, mature, and overmature, respectively.

### 2.4 Experimental procedure

A total of 157 samples were used to measure their impact response and maturity indices. The test was conducted every 5 days in a period of 20 days. In each test, 30–32 samples were used for the measurement. Kiwifruit maturity, including firmness, SSC, TA, and sensory score, was evaluated immediately after the impact response was measured.

Meanwhile, a test was conducted to evaluate the influence of impact on the fruit. In the same test day, additional 12 samples were divided into 3 groups. Each group was dropped onto the impact plate from the height of 2, 4, and 6 cm, respectively. After 24‐hr storage in the laboratory at about 20°C, such kiwifruits were used to measure firmness and cut for visual inspection (Ragni et al., 2010; Zhang et al., 2015).

### 2.5 Statistical analysis

#### 2.5.1 Quantitative analysis

A total of 15 vibration parameters were extracted in this study. First, the stepwise multiple linear regression (SMLR) method and the principal component analysis (PCA) were applied to reduce the dimension of input factors (Dong, Ni, & Kokot, 2013; Geesink et al., 2003; Liu, Sun, & Ouyang, 2010). PCA was carried out to extract information from the 15 vibration parameters, and principal components (PCs) which can explain more than 85% of the total variance were used for further analysis (Ibrahim et al., 2011; Kontogianni et al., 2010). Then, the stepwise multiple linear regression (SMLR) method and the back‐propagation neural network (BPNN) and PCA‐BPNN were applied to quantitative analysis of kiwifruit maturity. BPNN has a feedforward network structure including input, hidden, and output layers (Dong et al., 2013). In order to reduce the training time, only one hidden layer was used. Kiwifruit maturity was used as neurons of network output layer. The neurons of network input layer for the BPNN model were selected by the SMLR method, and that for the PCA‐BPNN model were PCs.

The performance of models was evaluated by the root mean square error of calibration (RMSEC), root mean square error of prediction (RMSEP), and correlation coefficients between the prediction values and actual values for the calibration and validation sample sets (*r*_c_ and *r*_p_).

#### 2.5.2 Qualitative analysis

Kiwifruits were categorized into immature, mature, and overmature groups. The BPNN and PCA‐BPNN models were used to distinguish kiwifruits with different maturities. Besides, the Fisher's discriminant analysis (FDA) was also carried out. The input variables for the FDA and BPNN models were selected by the SMLR method, and that for the PCA‐BPNN model were PCs.

## 3 RESULTS AND DISCUSSION

### 3.1 Results of possible mechanical damage test

After 24‐hr storage in the laboratory at about 20°C, no visible damage was found on the skin surface and flesh of the kiwifruits dropped from the height of 2 and 4 cm. From the height of 6 cm, slight damage was found in some samples in day 20. The results of puncture test showed that the firmness of these slight damaged samples was less than 4 N. The results can provide a reference for the determination of drop height.

### 3.2 Changes in the vibration parameters

**Figure 3:**
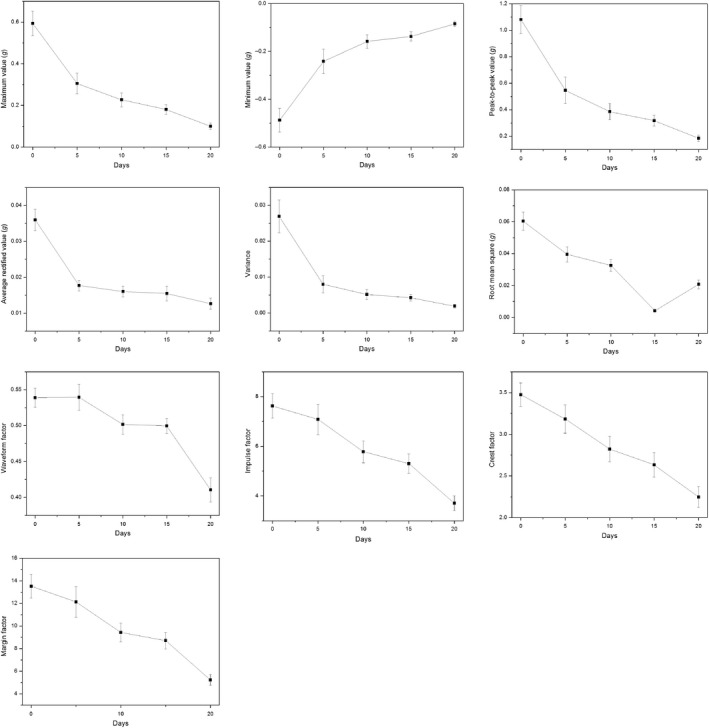
Time‐course changes in vibration parameters of kiwifruit during storage. Vibration parameters were extracted from the response signal obtained from the drop height of 6 cm. The bars represent the standard error

Figure 3 shows the time‐course changes in some vibration parameters (drop height of 6 cm) of kiwifruit during storage. All these vibration parameters in Figure 3 were on a decreasing or increasing trend during the entire storage period. Similarly, Taniwaki et al. (2009) and Zhang et al. (2014) used the LDV method to determine the ripeness of persimmons and pears, because the elasticity index declined gradually during storage. The results preliminarily indicated that the proposed method in our study can also be used to determine kiwifruit maturity. Other vibration parameters, including the signal duration, mean value, waveform area, skewness, and kurtosis, did not show an evident variation trend (not shown in the figure). The results of response signals obtained from the drop heights of 2 and 4 cm were basically the same.

### 3.3 Correlation between the vibration parameters and kiwifruit maturity

Table 3 shows the Pearson's correlation coefficients between the kiwifruit maturity indices and vibration parameters extracted from the response signal obtained from the drop height of 6 cm. The vibration parameters, except mean value and skewness, were well correlated with kiwifruit maturity indices. The results obtained from the drop heights of 2 and 4 cm were basically the same. Good correlations showed that the impact method used in this study had the potential for kiwifruit maturity evaluation. In addition, firmness and sensory score had better correlations with the vibration parameters than SSC and TA. This was because the vibration characteristics were directly related to fruit firmness, but were indirectly related to SSC and TA.

**Table 3:** Pearson's correlation coefficients between the vibration parameters and maturity indices (drop height of 6 cm)

	Firmness	Soluble solid content (SSC)	Titratable acidity (TA)	Sensory score
Maximum value	0.628**	−0.428**	0.345**	0.758**
Minimum value	−0.613**	0.333**	−0.365**	−0.733**
Signal duration	0.677**	−0.367**	0.401**	0.337**
Waveform area	0.587**	−0.317**	0.353**	0.686**
Mean value	−0.083	0.040	−0.123	−0.127
Peak‐to‐peak value,	0.653**	−0.453**	0.386**	0.753**
Average rectified value	0.637**	−0.304**	0.382**	0.731**
Variance	0.577**	−0.260*	0.108	0.678**
Root mean square	0.726**	−0.384**	0.296**	0.712**
Waveform factor	0.624**	−0.357**	0.326**	0.576**
Impulse factor	0.637**	−0.311**	0.337**	0.621**
Crest factor	0.706**	−0.326**	0.305**	0.638**
Margin factor	0.651**	−0.310**	0.371**	0.623**
Kurtosis	0.529**	−0.259*	0.303**	0.479**
Skewness	−0.020	0.045	−0.132	−0.036

* *p* < .05.

** *p* < .01.

### 3.4 Quantitative models of maturity evaluation

Initially, quantitative analysis of kiwifruit maturity was carried out. Samples were first divided into calibration and validation sample sets with a ratio of 3:1. Table 4 shows the results of quantitative analysis of kiwifruit maturity indices by the SMLR, BPNN, and PCA‐BPNN methods.

**Table 4:** Results of quantitative analysis of kiwifruit maturity indices by different modeling methods

Modeling method	Height (cm)	Firmness (*N*)				SSC (°Brix)				TA (%)				Sensory score			
		*r*_c_	RMSEC	*r*_p_	RMSEP	*r*_c_	RMSEC	*r*_p_	RMSEP	*r*_c_	RMSEC	*r*_p_	RMSEP	*r*_c_	RMSEC	*r*_p_	RMSEP
SMLR	6	0.784	3.024	0.701	4.144	0.428	2.734	0.360	3.042	0.363	0.032	0.339	0.033	0.831	1.518	0.785	1.667
	4	0.791	2.948	0.695	4.242	0.383	2.929	0.325	3.373	0.325	0.034	0.306	0.036	0.803	1.553	0.751	1.745
	2	0.734	3.845	0.646	4.647	0.328	3.237	0.294	3.524	0.332	0.034	0.307	0.035	0.786	1.817	0.728	1.994
BPNN	6	0.853	2.580	0.823	2.681	0.612	1.552	0.556	1.743	0.547	0.023	0.505	0.025	0.895	0.959	0.853	1.259
	4	0.848	2.647	0.821	2.606	0.589	1.673	0.502	1.978	0.504	0.026	0.473	0.028	0.896	0.956	0.849	1.236
	2	0.824	2.641	0.787	3.013	0.501	1.735	0.486	1.953	0.529	0.025	0.461	0.029	0.873	1.346	0.845	1.253
PCA‐BPNN	6	0.914	2.134	0.875	2.442	0.668	1.493	0.641	1.511	0.607	0.022	0.568	0.023	0.958	0.499	0.934	0.703
	4	0.925	2.028	0.881	2.359	0.678	1.506	0.624	1.522	0.530	0.024	0.494	0.026	0.961	0.504	0.935	0.693
	2	0.886	2.379	0.827	2.696	0.611	1.515	0.594	1.530	0.553	0.023	0.513	0.026	0.921	0.803	0.893	0.913

In the SMLR and BPNN models, different vibration parameters were selected for 3 drop heights by the SMLR method. Besides selected vibration parameters, fruit mass was added into the input variables. Acoustic vibration techniques give an overall measurement of the physical properties of fruit, including mass and internal structure (Abbott, Bachman, Childers, Fitzgera, & Matusik, 1968; Jancsok, Clijmans, Nicolai, & De Baerdemaeker, 2001). Therefore, mass is an important factor in the acoustic vibration measurement. The results showed that the BPNN model was better than the SMLR model. Compared with the PCA‐BPNN model, it was clear shown that the performance of BPNN model was further improved when PCs were used as input variables rather than a few vibration parameters selected by the SMLR method. The result was consistent with the finding of our former study (Zhang et al., 2015). This was because that more information of the tested sample was contained in PCs than a few vibration parameters.

In addition, better performance was obtained for firmness and sensory score evaluation. The results were consistent with the results of correlations between the vibration parameters and kiwifruit maturity indices obtained in part 3.3. The results obtained from 3 drop heights were close. However, the results obtained from the drop heights of 4 and 6 cm were a little better than that obtained from the drop height of 2 cm in most cases. This may be because the impact was too slight from a lower drop height. The optimized results for quantitative analysis of kiwifruit maturity were obtained by the PCA‐BPNN method. The optimized *r*_p_ (RESEP) for firmness, SSC, TA, and sensory score was 0.881 (2.359N), 0.641 (1.511°Brix), 0.568 (0.023%), and 0.935 (0.693), respectively. Such results indicated the feasibility of the proposed method for kiwifruit maturity evaluation. In most existing studies for fruit firmness detection by the acoustic vibration method, the *r*_p_ ranged from about 0.6 to 0.9 (Murayama, Konno, Terasaki, Yamamoto, & Sakurai, 2006; Taniwaki et al., 2009; Terasaki et al., 2001; Zhang et al., 2014). However, these results were obtained from the static state in most situations. The proposed method in our study provided a rapid way for online detection.

### 3.5 Qualitative models of maturity evaluation

The results of qualitative analysis of kiwifruit maturity by FDA, BPNN, and PCA‐BPNN methods are shown in Tables 5, 6, 7, respectively (drop height of 6 cm). Samples were also divided into calibration and validation sample sets with a ratio of 3:1.

**Table 5:** Discriminant results of kiwifruit maturity by the Fisher's discriminant analysis (FDA) method

Kiwifruit group	Predicted group membership							
	Calibration				Validation			
	1	2	3	Total	1	2	3	Total
1	35	5	2	42	13	2	0	15
2	0	34	6	40	0	8	3	11
3	0	14	22	36	0	5	8	13

**Table 6:** Discriminant results of kiwifruit maturity by the back‐propagation neural network (BPNN) method

Kiwifruit group	Predicted group membership							
	Calibration				Validation			
	1	2	3	Total	1	2	3	Total
1	39	3	0	42	13	2	0	15
2	1	37	2	40	0	9	2	11
3	0	7	29	36	0	2	11	13

**Table 7:** Discriminant results of kiwifruit maturity by the principal component analysis–back‐propagation neural network (PCA‐BPNN) method

Kiwifruit group	Predicted group membership							
	Calibration				Validation			
	1	2	3	Total	1	2	3	Total
1	40	2	0	42	14	1	0	15
2	0	37	3	40	0	10	1	11
3	0	2	34	36	0	1	12	13

In the FDA and BPNN models, the input variables were selected by the SMLR method. Similarly, *m* was added into the input variables. Discriminant results of BPNN models were obviously better than that of FDA model. Poor discriminant result of the FDA method was mainly caused by the misclassification of kiwifruits in group 3. As shown in Table 5, 14 samples (38.9%) and 5 samples (38.5%) in group 3 were misclassified into group 2 in the calibration sample set and validation sample set, respectively. The discriminant result was further improved when PCs were used as input variables in the PCA‐BPNN model instead of a few vibration parameters. The accuracy increased from 89.1% to 94.2% and from 84.2% to 92.1% in calibration and validation sample sets, respectively. In addition, most misclassification appeared in two neighboring groups. This was because that the maturity of some fruit was at the boundary of two groups, which led to some misclassifications between two neighboring groups by human sensory.

The results obtained from different drop heights are summarized in Table 8. Better discriminant results were also obtained from the drop heights of 4 and 6 cm, which was consistent with the result in quantitative analysis. Generally, good discriminant results proved that the proposed impact method can be used for kiwifruit maturity evaluation.

**Table 8:** Discriminant accuracy of different discriminant analysis methods for distinguishing kiwifruit maturity

Discriminant analysis method	Height (cm)	Accuracy	
		Calibration	Validation
FDA	6	77.3%	73.6%
	4	75.6%	71.0%
	2	75.6%	65.8%
BPNN	6	89.1%	84.2%
	4	89.9%	84.2%
	2	81.6%	78.9%
PCA‐BPNN	6	94.2%	92.1%
	4	92.5%	89.5%
	2	85.7%	84.2%

## 4 CONCLUSION

The impact method for kiwifruit maturity measurement was investigated in this study. The accelerometer was attached to the impact plate rather than the tested sample. Moreover, a 45‐degree inclination impact plate was used to avoid a second impact. The low‐cost design is quite appropriate for a rapid detection to meet the requirement of online detection. In addition, different models were established for quantitative and qualitative analysis of kiwifruit maturity. The PCA‐BPNN model showed the optimized results for the kiwifruit maturity evaluation. Such results indicated the feasibility of the proposed impact method for kiwifruit maturity evaluation. The procedure may also be applied to some other similar fruit.

## CONFLICT OF INTEREST

Wen Zhang declares that he has no conflict of interest. Aichen Wang declares that he has no conflict of interest. Zhenzhen Lv declares that she has no conflict of interest. Zongmei Gao declares that she has no conflict of interest.

## ETHICAL APPROVAL

All procedures performed in studies involving human participants were in accordance with the ethical standards of the institutional and/or national research committee and with the 1964 Helsinki Declaration and its later amendments or comparable ethical standards.

## INFORMED CONSENT

Informed consent was obtained from all individual participants included in the study.
